# Validation of the mean systemic filling pressure assessment with preserved arterial blood flow by comparing two methods of calculation

**DOI:** 10.1038/s41598-021-95350-7

**Published:** 2021-08-04

**Authors:** Roberto Alberto De Blasi, Stefano Finazzi

**Affiliations:** 1 Dipartimento di Scienze Medico-Chirurgiche e Medicina Traslazionale, Università degli studi di Roma Sapienza, Via di Grottarossa 1035, 00189 Rome, Italy; 2 Laboratorio di Clinical Data Science, Dipartimento di Salute Pubblica, Istituto di Ricerche farmacologiche Mario Negri IRCCS, Ranica, BG Italy

**Keywords:** Health care, Medical research, Pathogenesis, Signs and symptoms

## Abstract

We developed a method for measuring in vivo venular volumes and the mean systemic filling pressure in the limbs using near-infrared spectroscopy (NIRS). We aimed to validate the NIRS methodology by comparing two independent methods of calculation based on different physiological approaches. Pressure–volumes (P–V) curves were recorded following graded venous occlusion on the forearm. Values from a P–V curves analysis model (method 1) were compared with data derived from a resistor-capacitance calculation model (method 2) based on arterial pressure and venous compliance. We tested these methods on 10 healthy participants at rest and during exercise and on 6 severely ill patients. Results from method 1 were comparable with those calculated by method 2. Venular volumes calculated using method 1 correlated linearly with those calculated using method 2 both in participants (R^2^ = 0.98) and in patients (R^2^ = 0.94). A good agreement between methods was shown with few values out of the range of ± 1.96 standard deviation. Our findings added mathematical consistency for the NIRS methodology validation in the venular P–V assessment with no flow interruption. Further research will be required to confirm the relevance of the methodology in the clinical setting.

## Introduction

Blood volume and pressure in the postcapillary compartment (i.e., venules and small veins) are factors that determine venous return and systemic perfusion^1^. Pressure in this vascular compartment acts both as an upstream force moving blood back to the heart and as a force shifting fluids outside the microvascular bed. Consequently, post-capillary monitoring and management could be a useful tool to guarantee venous return and limit extravascular fluid leakage, particularly during diffuse inflammation.

Since the late 80 s, some authors have made efforts to obtain in vivo measurements of the mean circulatory filling pressure (mcfp), for use as indicator of the average integrated pressure throughout the circulatory system. These methods require an absence of blood flow for calculating the vascular pressure variables and this was obtained by hindering venous return to the heart and extrapolating to zero the resulting blood flow reduction, or by creating a stop-flow in the upper arm^2,3^.

Unfortunately, these methods have several relevant limitations. First, inducing mechanical changes in the cardiac output (CO) is cumbersome and requires patients who are mechanically ventilated. Second, the extrapolation of CO to zero flow assumes a forced linearity in the pressure–volume (*P–V*) relationship that underestimates the intravascular pressure^3,4^.

Additionally, despite these methods allow to estimate the vascular system filling, stopping the flow, they do not provide knowledge on the upstream pressure for venous return (VR), thus omitting a crucial determinant of the cardiovascular system function.

Previously, we had developed a method for measuring in vivo pressures and volumes in venules and small veins in the limbs based on the variation in light absorption using near-infrared spectroscopy (NIRS) after downstream venous occlusion, thus obtaining a measurement of the upstream pressure for venous return, without interrupting the arterial blood flow^5^. This pressure, called mean systemic filling pressure (MSFP) according to a definition by Magder^1^, is often erroneously considered as a synonym of the mcfp, but, although quite similar, it differs from the mcfp because it is only related to the volume in the systemic vein.

The method we developed complies with the principles of strain gauge plethysmography and NIRS, and it has the advantage of measuring microvascular bed volume and its subdivisions: stressed and unstressed volumes (*V*_s_, *V*_u_). Given that a direct measurement of post-capillary pressures and volumes cannot be obtained in clinical conditions, we derived these measurements using a model based on the analysis of the *P–V* curves^5^. We applied it in two clinical conditions by obtaining data that conform to pathophysiology^6,7^. Unfortunately, this method has not yet been fully validated because no gold standard exists and limitations of previous methods do not allow us to consider them as a reference.

In this study, we aimed to supply mathematical derivations from well-established physiological basics equations to validate the NIRS methodology, thus adding inferential elements to our previous construct. To do so, we calculated the MSFP and venular volume with a resistor-capacitance calculation model based on arterial pressures and venous compliance and compared results with those obtained by the previous *P–V* curves analysis, reflecting different P–V arrangements of the microvascular bed. As these models were based on two independent calculations reflecting two different physiological approaches to VR, finding correlations between models’ results could provide mathematical consistency to the NIRS methodology we used.

## Methods

The study was conducted at Sant’Andrea University Hospital, Rome, Italy. We performed multiple measurements on 10 healthy volunteers within the hospital personal (3 men and 7 women), with an average age of 26.5 ± 2.2 years and a body mass index of 21 ± 2.2 kg/m^2^ (range: 19–26), and on 6 critically ill patients consecutively admitted to the Intensive Care Unit in 2018. The patients’ average age was 58.7 ± 19.0 years and the body mass index was 26.4 ± 2.4 kg/m^2^ (range: 22.86–29.39). For this study, we considered 24 measurements from healthy participants and 18 measurements from patients as representing the healthy adult population and patients with an impaired microvascular function. The investigative procedures have been performed in accordance with the Declaration of Helsinki of 1975, as revised in 1983, and the research has been approved by the ethics committee of the Sapienza University of Rome at the Sant’Andrea University Hospital the hospital (Prot. 7715/13, 7173/13). All participants or their next of kin provided written informed consent before the start of the study.

### NIRS settings and data analysis

We derived the forearm microvascular bed volumes from the sum of oxygenated hemoglobin/myoglobin (oxyHb/Mb) and deoxygenated Hb/Mb (deoxy Hb/Mb) μM concentrations ([Hb]tot/[Mb]tot) measured using a NIMO-4 continuous-wave photometer (Nirox srl, Brescia, Italy)^8^. Then, we calculated the blood volume per 100 mL of tissue after accounting for the mean corpuscular hemoglobin concentration, mean corpuscular volume, and hematocrit levels in each participant. Since the Hb in arterioles accounts for only 3% of the NIRS signal, and because these vessels as well as capillaries are poorly distensible^9^, the changes in [Hb]tot during venous compression represented almost exclusively the changes that occurred in the venules and small veins. Data were acquired at a sampling time of 0.1 s, thus facilitating analysis of rapid changes in the [Hb] levels with a dedicated NIMO software program.

### Protocol

The red blood cell count, cell volumes and the mean corpuscular hemoglobin content were obtained for each participant. To avoid unnecessary insertion of arterial catheters, in healthy participants the arterial blood pressures was recorded non-invasively by a pneumatic cuff, whereas in patients we used data from the invasive devices they had for cardiovascular monitoring. The choice of allowing two different technologies for measuring the arterial pressure was made as, in the range of pressure we studied, the mean arterial pressure was shown to differ little between the invasive and non-invasive approach^10^. In addition, arterial pressure does not interfere with blood volume changes due to venous occlusions^11^.

We performed measurements in the dominant forearm and positioned a NIRS probe at the top of the flexor digitorum superficialis (FDS) muscle and verified the correct placement of the probe visually. A pneumatic cuff was placed around the arm 5 cm proximal to the antecubital crease, with its tube connected to an automatic inflation system (model E-20, Hokanson Rapid Cuff Inflator and AG101 Air Source; PMS Instruments Ltd, Maidenhead, UK) capable of reaching a manually predefined pressure in less than 0.5 s. To ensure that there were no differences in hydrostatic pressure due to the position of the forearm during measurement and that no correction factor was needed, we restrained the forearm in a plastic frame at an angle of 135° in relation to the upper arm and placed the NIRS probe at the same level as the right atrium^12^. The study took place in temperature-controlled rooms, and participants were made to lie down in a comfortable position. To extend the range of values, we experimentally increased the extravascular pressure by graded muscle isometric contractions in healthy participants.

In this study, unlike in previous ones^5–7^, we first inflated the cuff to 50 mmHg for 120 s, according to Halliwill’s technique^13^, to ensure equilibrium between the cuff pressure (*P*_cuff_) values and intravascular pressure (*P*_i_) and achieve their equivalence^14^. Then we decreased *P*_cuff_ by 5 mmHg down to 0 mmHg, with each step lasting 20 s (10 data points). The five [Hb]tot values preceding each *P*_cuff_ drop of 5 mmHg were manually averaged into 1 value from 50 to 0 mmHg, obtaining 10 steady volume values to generate *P–V* curves. In both methods, it is believed that applying a pressure P with a cuff to the arm generates an extra downstream pressure and blood volume increase that overcomes the baseline venule pressure (*P*_0_) (i.e. the mean systemic filling pressure).

Healthy participants were asked to perform maximal voluntary contraction (MVC) with a digital hand grip (Kern & Sohn GmbH, Balingen, Germany) recording the force in kilograms.

After a first set of measurement at rest, two additional sets of measurements were performed during isometric exercises by clenching the handgrip at 10% and at 20% MVC, and maintaining the force for 3 min. The isometric muscle contractions were separated by a resting period of 3 min. During contractions, the cuff was inflated to a pressure of 50 mmHg and then deflated following the same framework used for the first set of measurements performed at rest.

Measurements were performed on patients at rest. All patients received treatments established by the doctor on charge and NIRS measurements were performed before and after changes in vasoactive agents infusion rate or fluid administration.

### Calculation of MSFP and venular volumes

Both methods are based on the cuff pressure and venular volume measurements. Assuming that these variables are correctly measured, we compared two independent methods of calculation to derive the MSFP, venular volume partitioning (V_u_, V_s_) and venular compliance.

#### Method 1

**Figure 1:**
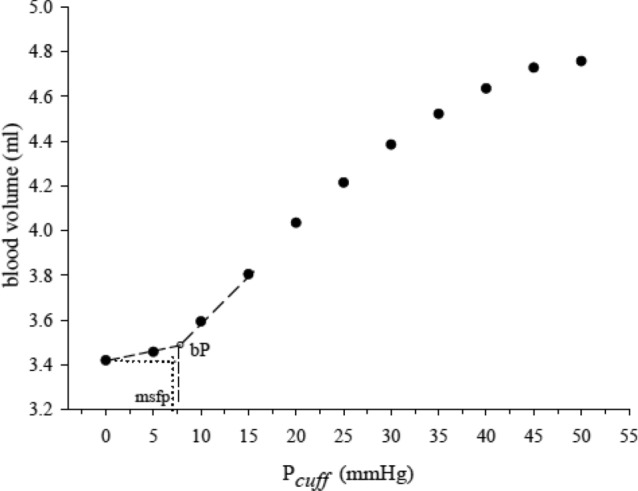
A representative pressure–volume curve. Mean systemic filling pressure: mspf, breakpoint pressure: bP.

Preliminary measurements performed by us showed three slopes of the P–V curve, in the range of the *P*_cuff_ used in this study, that we considered for the MSFP and compliance calculations (Fig. 1).

Based on the evidence that venous compliance is not linear^5,15^, we examined pressure-compliance changes with a multi-linear model. We interpreted the upper part of the *P–V* curve as venular stretching (venular compliance), the intermediate and steeper part as vascular bed recruitment induced by the increase in *P*_i_, and the lower portion of the curve as blood volume changes that occur at a *P*_i_ lower than that needed to recruit the venular bed.

To avoid any assumption about the shape of the P–V curve, the relationship was calculated as the numerical derivative (2-point difference derivative) of each data point using the following equation:

$$C\left(Pi\right)= \frac{V{\text{i}}+ \text{1} -V{\text{i}}}{P{\text{i}}+ \text{1} -P{\text{i}}}$$

where 0 < *P*_i_ < 50 mmHg, C is compliance, *P* is intravascular pressure, *V* is blood volume*,* and i is an index value from 1 (corresponding to 0 mmHg) to 10 (corresponding to 45 mmHg). In this model, the breakpoint pressure (*bP*), which separates the intermediate phase from the low-pressure phase, was determined by the *P*_i_ corresponding to the highest value of compliance. To calculate MSFP, we extrapolated the measurements of the steeper *P–V* segment using linear regression from the *bP* to the blood volume measured before occlusion (baseline volume, *V*_0_) on the pressure axis. This corresponds to the *P*_i_ within venules and small veins in the presence of blood flow and was thus deemed to represent the MSFP.

In order to partition the V_0_ in Vs and Vu, we calculated Vs as the volume included in the MSFP taking into account the steeper P–V segment from the bP^7^.

#### Method 2

As NIRS makes no distinction between [Hb] and [Mb], we assumed that at rest, Mb is totally oxygenated^16^ and that the oxygen saturation of [Hb] at baseline corresponds to that measured following venous occlusion at a low P_*cuff*_. Since light absorption at baseline is the result of deoxy-[Hb] plus the percentage of [HbO_2_] in tissue blood, we could calculate the [MbO_2_] contribution to V_0_. Then, we obtained Vu by subtracting [MbO_2_] contribution from Vs^7^.

**Figure 2:**
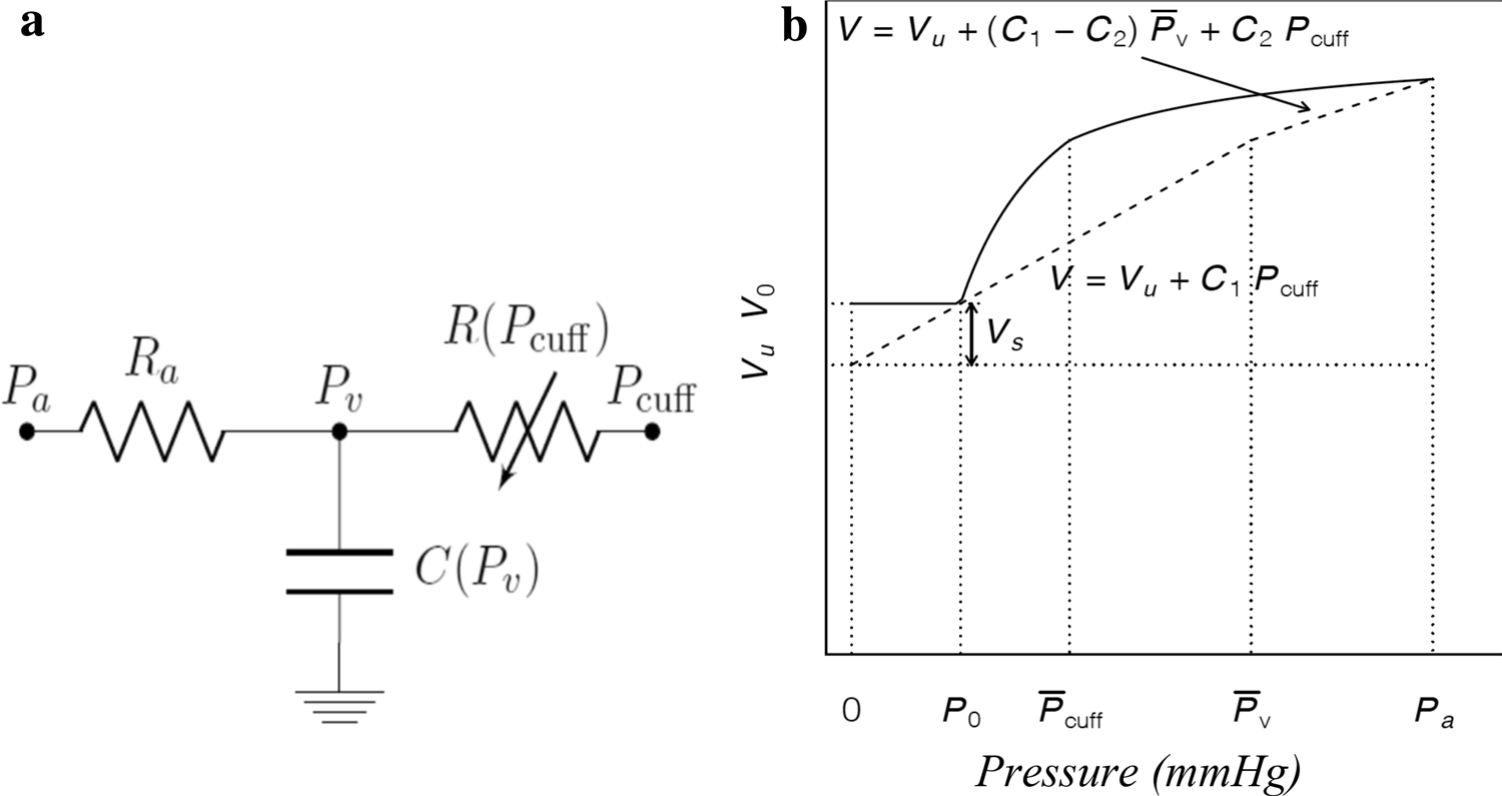
(**a**) An electric scheme of the resistor-capacitance model. *P*_*a*_ and *P*_*v*_ are the arterial and venular pressure. *P*_cuff_: cuff pressure, *C*: venular compliance, *R*_*a*_: total resistance upstream of venules, *R(P*_cuff_*)*: the variable resistance generated by the cuff pressure. (**b**) Venular volume as a function of the cuff pressure. The volume is constant for *P* < *P*_0_. For *P* > *P*_0_ it grows according to the equations derived from the model picture in Fig. 2a (see SM for the derivation). *C*_1_: compliance associated with recruitment of the microvascular bed, *C*_2_: compliance associated with vessel elasticity, *V*: venular volume (ml), *V*_*0*_*:* venular volume at baseline (ml). *V*_*u*_ and *V*_*s*_: unstressed and stressed volume (ml).

For this method, we developed an analog resistor–capacitance model of the vascular system^17^, by adapting the model proposed by Van Vo et al.^18^. As an analogy with the electric scheme, the current, difference in electric potential, resistances, and capacitance represented blood flow, pressure difference, vascular resistance, and vascular compliance, respectively (Fig. 2a).

We modeled the vascular compartment between the arteries and veins upstream of the pneumatic cuff. The initial and final pressures on our circuit were represented by the arterial pressure *P*a and *P*_cuff_. We defined *R*_a_ as total resistance in the upstream venules, *C* as venular compliance, *R* as venular resistance and *P*_*v*_ as venular pressure. In our model, both *R* and *C* depended on the P_cuff_ because of venular pressure transmission. In particular, *R* equals the baseline value when the P_cuff_ is lower than the MSFP value and grows linearly when P_*cuff*_ rises above it. In our model, up to a certain *Pv* value, *C* reflects the recruitment of the microvascular bed (*C*_1_). Above this *Pv* value, the vascular bed is fully recruited and venular compliance (*C*_2_) reflects the vessel wall elasticity.

Based on this premise and by assuming the blood flow upstream and downstream venular compartment in the steady state as a constant, we derived a relationship between the *V*_0_ and *P*_cuff_, which is reported in Fig. 2b. All measurements were taken when the blood flow in the venule section had reached the stationary state after each change in the cuff pressure. Accordingly, we solved the circuit equations in the late-time stationary limit, when the transient phase had ended. When the *P*_*cuff*_ is lower than the MSFP (*P*_0_) venular volume equals the sum of *V*_u_ and *V*_s_, which is the baseline volume (*V*_0_), namely, the volume attained when the *P*_cuff_ is not applied. Above *P*_0_, venular volume increases as a result of the *P*_cuff_ values increase, showing two different slopes corresponding to the vascular bed recruitment and vessel wall elasticity. Calculations and relationships between the variables and details on the model are reported in supplementary methods S1.

Variable values calculated with the model fitted with NIRS were analyzed using generalized nonlinear regression with R software (version 1.1.414, 2009–2018 RStudio, Inc.).

### Statistical analysis

From preliminary results comparing P–V values calculated by the two methods, a slope of the linear regression line of 0.95 and 0.97 respectively with a standard deviation (SD) of the regression errors of 0.88 and 0.17 were showed for patients, whereas for healthy participants a slope of 0.70 and 0.98 with a standard deviation of 0.91 and 0.18. These data entail that a sample size of 4 measurements for patients and 10 measurements for healthy participants would be adequate to detect a difference between methods in the order of magnitude previously assessed, with a statistical power of 80% and an alpha error of 0.05 (PSSize Calculations software, version 3.0; Informer Technologies).

All data were expressed as mean ± SD. The Kolmogorov–Smirnov test was used to assess normal data distribution. We used the analysis of variance (ANOVA) for repeated measurements for testing values differences within the same group. The Bland and Altman test was used to compare the two methods of calculation plotting the differences between data measured by method 1 and 2 and against the averages for the two methods. We calculated the mean difference between two methods of measurement (the “bias”), and 95% limits of agreement as the mean difference (± 1.96 SD). Pearson’s correlation coefficient (r) and regression analysis were used to test data linearity of the two methods. A *p*-value less than 0.05 was considered to indicate statistical significance. Data were analyzed with the software program MedCalc (version 11.5; MedCalc Software, Ostend, Belgium).

## Results

### Characteristics of participants

Of the 10 healthy participants, 7 had measurements at baseline, at 10% and 20% MVC. Two participants were excluded from measurements at 20% MVC because of an unreliable NIRS signal, and for 1 participant we only included the measurement at baseline. We performed 18 measurements on the six patients (2 with trauma, 4 with sepsis), before and after all changes in fluid balance, norepinephrine infusions, or airway pressures.

**Figure 3:**
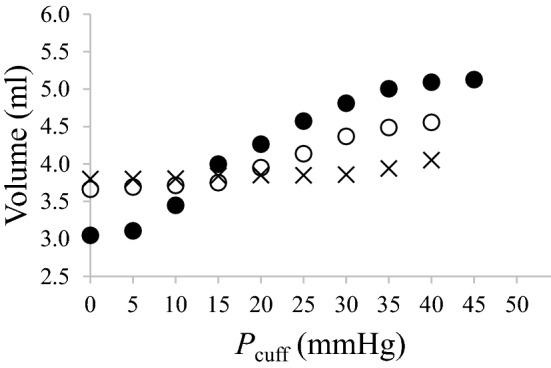
Representative pressure–volume curves measured at rest (filled circles), at 10% (open circles) and 20% (x signs) of maximal voluntary contraction.

All participants at rest and the patients showed a distinct shape illustrating the P–V relationship, with three different slopes and two possible inflection points. Gradual isometric exercises led to a flattening in the shape of the *P–V* relationship (Fig. 3).

### Measurements in healthy participants

At rest, the MSFP calculated in healthy participants by method 1 yielded values higher than those reported in our previous studies (6.9 ± 2.3 mmHg vs 4.2 ± 0.5 mmHg, *p* < 0.001 or 4.9 ± 2.4, *p* = 0.040). In contrast, the volume values matched those previously reported^7^. ANOVA showed significant changes in MSFP (*p* < 0.001) when comparing sets of measurements at rest and during exercise, showing a progressive increase in MSFP from rest to 10% and 20% MVC. The changes in the shape of the P–V curve due to exercise resulted in no change in volume (*p* = 0.440). Thus, calculations revealed a decrease in the Vs compliance (*p* = 0.010).

### Fit of methods 2

**Figure 4:**
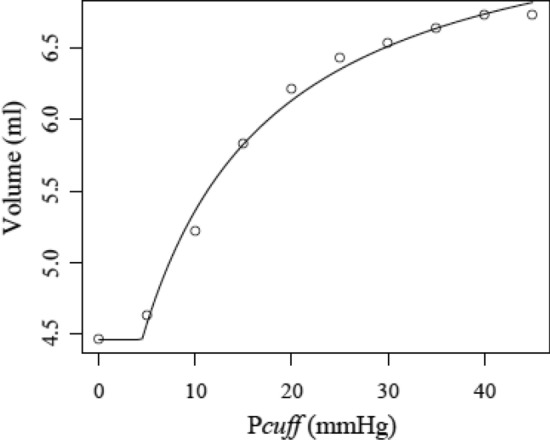
A representative pressure–volume curve on one participant in which the resistor-capacitance model (line) fits the pressure–volume data points (open circles) measured by the near infrared spectroscopy. Below the MSFP, the venular volume remained constant, whereas above it, the P–V curve showed initially small volume changes, a second response with a higher compliance, corresponding to vascular bed recruitment, and third response with a lower compliance, corresponding to the stretch of the venular wall, as expected.

Both methods gave rise to similar P–V curves when P_cuff_ decreased. Our mathematical model (model 2) qualitatively and quantitatively reproduced the values obtained by NIRS (Fig. 4).

### Agreement between methods 1 and 2

**Figure 5:**
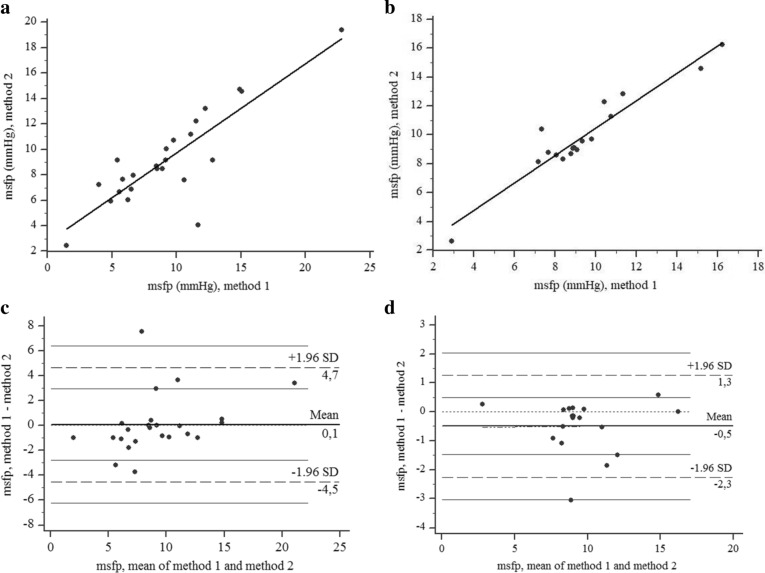
(**a**) Regression line of MSFP values measured with method 1 and 2 in healthy participants. The linear regression is described by the equation: y = 2.73 + 0.70 x. (**b**) Regression line of MSFP values measured in patients. The linear regression is described by the equation: y = 0.98 + 0.95 x. (**c**) The Bland–Altman test comparing the MSFP values measured with method 1 and 2 in healthy participants. (**d**) The Bland–Altman test comparing the MSFP values measured in patients.

The mfsp values calculated using methods 1 and 2 showed a linear correlation both in healthy participants, for data at rest and during contractions (*R*^2^ = 0.72, *p* < 0.001), and in patients (*R*^2^ = 0.91, *p* < 0.001) (Fig. 5a,b).

The two regression lines showed different values for intercepts and slopes, with values concentrated mainly within a small range in both groups of participants. The Bland–Altman test selected one value out of those in the range of ± 1.96 SD for healthy participants and one for patients (Fig. 5c,d).

Measurements of the volumes in venules (*V*_u_ + *V*_s_) showed a linear correlation between values calculated using methods 1 and 2, both in healthy participants (*R*^*2*^ = 0.98, *p* < 0.001; y = 0.04 + 1.0x) and in patients (*R*^*2*^ = 0.94, *p* < 0.001; y = 0.05 + 0.97 x).

**Figure 6:**
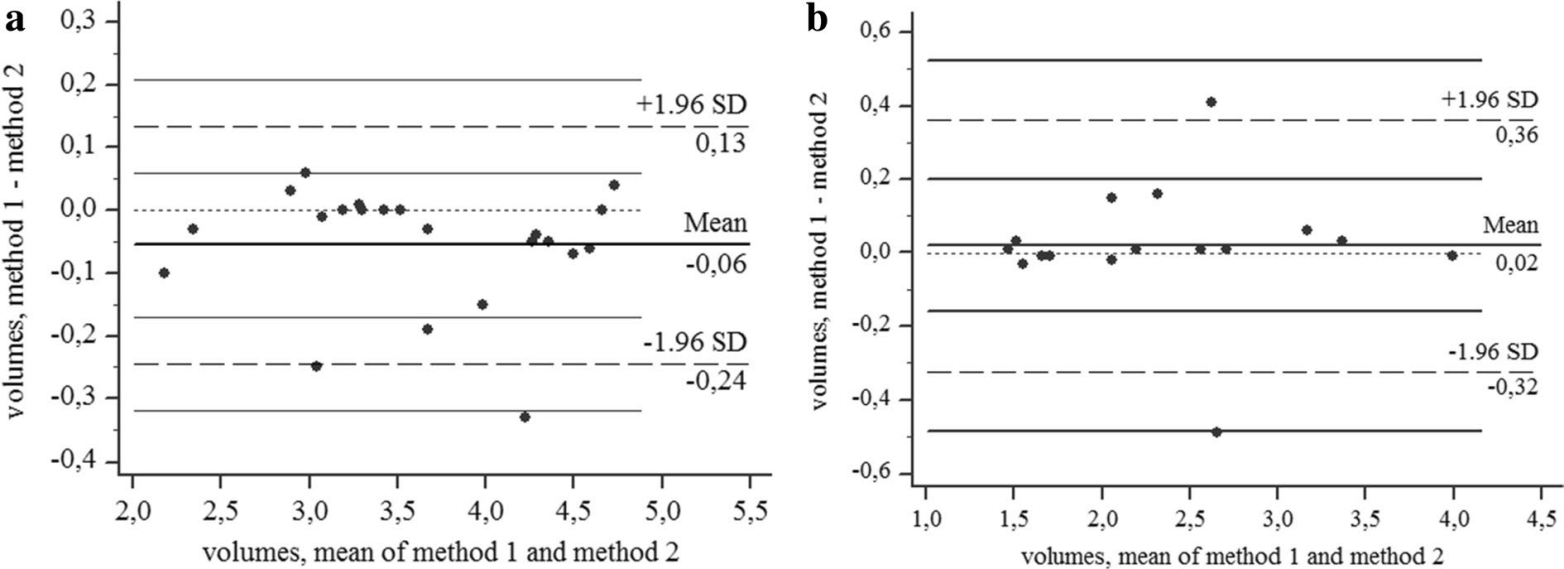
(**a**) The Bland–Altman test comparing volume values measured with method 1 and 2 in healthy participants. (**b**) The Bland–Altman test comparing volume values measured in patients.

Intercepts and slopes of the two regression lines were similar, and values were homogenously distributed across a wide range in both groups of participants. The Bland–Altman test displayed two values out of ± 1.96 SD for healthy participants and two for patients (Fig. 6a,b).

## Discussion

From our study findings, venular pressures and volumes derived from two independent calculation models showed similar values, adding rigorous mathematical and physiological elements for the NIRS method validation.

This investigation originates from the need to validate a measurement technique in the absence of a well-founded in vivo benchmark. In a previous study, we compared NIRS measurements of MSFP in the forearm, based essentially on method 1, with those obtained by evaluating blood volume changes using strain-gauge plethysmography^5^. Although we have added an innovative method for calculating postcapillary the blood volume partitioning, this paper is not a proof that the NIRS method provides the correct value of the venular volume, whose validity is discussed elsewhere^8^.

Validation is a complex concept that requires defining a priori and explicitly the concept of what we want to demonstrate^19^. In this case, the construct consists of the hypothesis that requirements on which the NIRS methodology calculation is based cannot be accidental, and consequently can be considered valid if they conform to well-known physiological principles with regard to microcirculation.

The values of MSFP and venular volumes obtained by our NIRS methodology, in contrast to those of the mcfp obtained with the arterial flow limitation maneuvers, conform to the physiologic knowledge on venous return and the hydrostatic pressure^1^. A validation framework also needs to determine which empirical available evidence links data to the meaning we give to it. In order to provide evidence supporting the variables that reflect the physiological meaning we attribute to them, we tested NIRS measures at rest and in an experimental condition in which rapid pressure changes within the muscular tissue were induced. Evidence showed a good correlation between muscle force under both active and passive conditions, and intramuscular pressure (IMP)^20,21^. Since IMP is typically identified as a measurement of fluid pressure, including the contribution of the interstitial fluid^22^, it is reasonable to suppose that a force-related IMP increase affects pressure inside venules. This simulates an interstitial fluid pressure higher than normal, as observed during inflammation. Our results confirmed the incremental MSFP values by varying muscle efforts, providing evidence of the sensitivity of NIRS technology to changes in interstitial pressure.

Higher MSFP values measured at rest in healthy participants by method 1 when compared to those previously reported can be explained by a small change we introduced in the calculation of method 1. It included the estimation of the lower part of the P–V curve, the low-pressure phase. In this revised method, we extrapolated the steeper P–V segment in the intermediate phase to the pressure axis. The rationale behind this change relies on the reasoning that P–V data, resulting from the change in vascular compliance at the beginning of vascular bed recruitment, should reasonably reflect the force that the vascular bed exerts to oppose its recruitment. We were able to verify in monitored patients that this new MSFP calculation gave rise to MSFP—central venous pressure gradients in accordance to the patient’s patho-physiologic condition^1^.

The lower R^2^ observed in the MSFP overall values of healthy participants and patients could be justified by the flattering of the curve shapes during muscle contractions, which could have a significant impact on the calculation by the two methods. On the contrary, patients at rest despite having a wider range of MSFP and volume values, showed a more homogeneous curve shape.

It is important to clarify the available evidence that justifies the utility and relevance of the data. The answer is derived from the results of two studies in which the NIRS methodology was applied in critically ill patients and in those who had undergone cardiac surgery^6,7^. In these studies, the evaluation of changes in the venular compartment was crucial for understanding the effects of volume loading and extracorporeal pump on tissue perfusion. In addition, we collected unpublished data on patients with trauma or sepsis that highlighted the importance of in vivo monitoring of the venular compartment for comprehending the effects of treatments.

Another critical issue comprised determining the consequences of data errors in terms of clinical implications. Although no measurement can be completely free from uncertainties and errors, the possibility of incorrectly recording venular pressures and volumes may theoretically lead to consideration of giving fluids or vasoactive agents to a patient when it is not necessary or, on the contrary, refraining from giving them when needed. However, as venular values vary according to variables measured in macrocirculation, the validity of NIRS variables results from their consistency with data from the macrocirculation as well as from changes in therapy. An effective way to account for possible errors in measurements is to look at the shape of the *P–V* curve. The final consequential element of construct validation is determined by judging the social acceptability of consequences that occur as a result of using a study’s findings, measures, or inferences. To fulfill this last point, we need to conduct further research and perform studies that possibly demonstrate the validity of NIRS variables in different contexts of clinical practice.

Our study has several limitations. The first possible limitation is that we compared the total venular volume with the two methods without categorizing it into *V*_s_ and *V*_u_. This relates to the inability to extrapolate the *V*_u_ using method 2. Another limitation is the possible inaccuracy in the absolute *V*_u_ value calculated using method 1, due to the overlapping light absorption spectra of Hb and Mb. This makes identification of Mb non-feasible. Notwithstanding, the percentage of the NIRS signal due to Mb in the skeletal muscle is lower and contributes modestly to the calculation of *V*_u_ using method 1. Additionally, since the Mb concentration and its saturation remained unchanged over several days, we considered the *V*_u_ changes in the same participant to be reliable. In order to collect more data for the *P–V* curve, we performed measurements with a 2-mmHg drop. However, because of the difficulty in manually setting such a small variation in the *P*_*cuff*_ and obtaining stable [Hb] values, we excluded these measurements from the results in view of potential inaccuracies.

## Conclusion

In this study, we added mathematical consistency to our previous construct, based on P–V curves analysis, to validate with two independent methods of calculation the NIRS methodology for the assessment of venular bed without flow interruption. The methodology was tested in healthy volunteers at rest and during exercise and in patients and variables results were in conformance with the physiological principles with regard to microcirculation, as hypothesized. Further research will be required to confirm the utility and relevance of this method in cardiovascular diagnosis and treatment.

## Supplementary Information

Supplementary Information 1.

Supplementary Information 2.

## Data Availability

The whole dataset generated during and/or analyzed during the current study are available from the corresponding author on reasonable request.
