# Supplementary material for: Validation of the mean systemic filling pressure assessment with preserved arterial blood flow by comparing two methods of calculation
Source: Sci Rep. 2021 Aug 4;11:15844. doi: 10.1038/s41598-021-95350-7 (PMC8338930; doi:10.1038/s41598-021-95350-7)
Supplement: Supplementary file 1 — Supplementary Information 1. [file 41598_2021_95350_MOESM1_ESM.pdf]

Derivation of Eqs. (4) and (8)

Replacing

$$R(P_{\text{cuff}}) = R_0 \left(\frac{P_{\text{cuff}}}{P_0} - 1 \right) \tag{1}$$

into

$$P_v = P_{\text{cuff}} + \frac{R(P_{\text{cuff}})}{R_a + R(P_{\text{cuff}})} (P_a - P_{\text{cuff}}), \tag{2}$$

we obtain

$$P_v = P_{\text{cuff}} + \frac{R_0}{R_a + R_0(P_{\text{cuff}}/P_0 - 1)} (P_a - P_{\text{cuff}}) \left(\frac{P_{\text{cuff}}}{P_0} - 1 \right). \tag{3}$$

Adding and subtracting P_0:

$$P_v = P_0 + P_0 \left(\frac{P_{\text{cuff}}}{P_0} - 1 \right) + \frac{R_0}{R_a + R_0(P_{\text{cuff}}/P_0 - 1)} (P_a - P_{\text{cuff}}) \left(\frac{P_{\text{cuff}}}{P_0} - 1 \right). \tag{4}$$

Collecting $P_{\text{cuff}}/P_0 - 1$,

$$P_v = P_0 + \left(\frac{P_{\text{cuff}}}{P_0} - 1 \right) \left[P_0 + \frac{R_0}{R_a + R_0(P_{\text{cuff}}/P_0 - 1)} (P_a - P_{\text{cuff}}) \right]. \tag{5}$$

Summing the terms in the square brackets

$$P_v = P_0 + \left(\frac{P_{\text{cuff}}}{P_0} - 1 \right) \left[\frac{R_a P_0 + R_0 P_{\text{cuff}} - R_0 P_0 + R_0 P_a - R_0 P_{\text{cuff}}}{R_a + R_0(P_{\text{cuff}}/P_0 - 1)} \right], \tag{6}$$

from which

$$P_v = P_0 + \left(\frac{P_{\text{cuff}}}{P_0} - 1 \right) \left[\frac{R_a P_0 - R_0 P_0 + R_0 P_a}{R_a + R_0(P_{\text{cuff}}/P_0 - 1)} \right]. \tag{7}$$

Dividing both numerator and denominator by R_a

$$P_v = P_0 + \left(\frac{P_{\text{cuff}}}{P_0} - 1 \right) \left[\frac{P_0 + (R_0/R_a)\,(P_a - P_0)}{1 + (R_0/R_a)(P_{\text{cuff}}/P_0 - 1)} \right], \tag{8}$$

which is Eq. (4) of the SM.

Defining

$$\beta = \frac{R_0}{R_a}, \tag{9}$$

replacing it into the above equation, and collecting P_0 in the second term

$$P_v = P_0 + P_0 \left[1 + \beta \left(\frac{P_a}{P_0} - 1 \right) \right] \left[\frac{(P_{\text{cuff}}/P_0) - 1}{1 + \beta(P_{\text{cuff}}/P_0 - 1)} \right]. \tag{10}$$

It is convenient to express the terms $P_a/P_0 - 1$ and $(P_{\text{cuff}}/P_0) - 1$ through the function f, defined as

$$f(x) = \frac{x}{P_0} - 1 \tag{11}$$

for $x > P_0$. Finally, defining the function r as

$$r(x) = \frac{f(x)}{1 + \beta\, f(x)}, \tag{12}$$

$$P_v = P_0 + P_0 \left[1 + \beta f(P_a) \right] r(P_{\text{cuff}}), \tag{13}$$

which is Eq. (8) of the SM.

Derivation of Eq. (28)

Starting from Eq. (27)

$$r(\bar{P}_{\text{cuff}}) = \frac{V_2 - V_0}{V_1 - V_3} \tag{14}$$

and

$$r(x) = \frac{f(x)}{1 + \beta\, f(x)}, \tag{15}$$

$$\frac{f(\bar{P}_{\text{cuff}})}{1 + \beta\, f(\bar{P}_{\text{cuff}})} = \frac{V_2 - V_0}{V_1 - V_3}. \tag{16}$$

We obtain

$$f(\bar{P}_{\text{cuff}}) = \left[\frac{V_1 - V_3}{V_2 - V_0} - \beta\right]^{-1}. \tag{17}$$

Using

$$f(x) = \frac{x}{P_0} - 1, \tag{18}$$

$$\frac{\bar{P}_{\text{cuff}}}{P_0} - 1 = \left(\frac{V_1 - V_3}{V_2 - V_0} - \beta\right)^{-1}, \tag{19}$$

$$\bar{P}_{\text{cuff}} = P_0 \left[1 + \left(\frac{V_1 - V_3}{V_2 - V_0} - \beta\right)^{-1}\right]. \tag{20}$$
