# Supplementary material for: Validation of the mean systemic filling pressure assessment with preserved arterial blood flow by comparing two methods of calculation
Source: Sci Rep. 2021 Aug 4;11:15844. doi: 10.1038/s41598-021-95350-7 (PMC8338930; doi:10.1038/s41598-021-95350-7)
Supplement: Supplementary file 2 — Supplementary Information 2. [file 41598_2021_95350_MOESM2_ESM.pdf]

Validation of the mean systemic filling pressure assessment with preserved arterial blood flow by comparing two methods of calculation

Supplementary Information: Derivation of the relationship between cuff pressure and venular volume

Roberto Alberto De Blasi[1] and Stefano Finazzi[2]

[1]Dipartimento di Scienze Medico-Chirurgiche e Medicina Traslazionale, Universit degli studi di Roma Sapienza via di Grottarossa 1035, 00189 Roma - Italy - roberto.deblasi@uniroma1.it
[2]Laboratorio di Clinical Data Science, Dipartimento di Salute Pubblica, Istituto di Ricerche Farmacologiche Mario Negri IRCCS, Ranica (BG), Italy - stefano.finazzi@marionegri.it

We define R_a as the total vascular resistance upstream the venules, P_a and P_v as the arterial and venular pressures and C as the venular compliance. By applying to the arm a pressure P_{cuff} with a cuff, an extra resistance $R(P_{\text{cuff}})$ is generated downstream when the cuff pressure overcomes the baseline venule pressure P_0 (mean circulatory filling pressure). A resistor-capacitance model of this system is pictured in Supplementary Figure 1. According to the experimental settings, each measurement of the volume of the venular compartment is performed in a stationary state, after the transient phase has ended. For this reason, we solve the system represented in Supplementary Figure 1 in the stationary late-time regime, neglecting transient time dependences.

Venular pressure – cuff pressure. By considering either the full circuit or the downstream section, the blood flow Q can be expressed as

$$Q = \frac{P_a - P_{\text{cuff}}}{R_a + R(P_{\text{cuff}})} = \frac{P_v - P_{\text{cuff}}}{R(P_{\text{cuff}})}. \tag{1}$$

The venular pressure is derived as a function of the cuff pressure P_{cuff} from Eq. (1),

$$P_v = P_{\text{cuff}} + \frac{R(P_{\text{cuff}})}{R_a + R(P_{\text{cuff}})}(P_a - P_{\text{cuff}}). \tag{2}$$

Since the resistance $R(P_{\text{cuff}})$ appears when the cuff pressure overcomes the baseline venular pressure P_0, we can approximate $R(P_{\text{cuff}})$ as

$$R(P_{\text{cuff}}) = R_0 \left(\frac{P_{\text{cuff}}}{P_0} - 1 \right), \tag{3}$$

for $P_{\text{cuff}} \geq P_0$ and $R(P_{\text{cuff}}) = 0$ for $P_{\text{cuff}} < P_0$. Furthermore, when the cuff pressure is lower than the baseline venular pressure ($P_{\text{cuff}} < P_0$), the venular pressure P_v equals P_0.

When $P_{\text{cuff}} \geq P_0$, it is convenient to express Eq. (2) in terms of $P_{\text{cuff}}/P_0 - 1$, using Eq. (3). After some lengthy manipulation, we obtain

$$P_v = P_0 + \left[P_0 + \frac{R_0}{R_a}(P_a - P_0) \right] \frac{P_{\text{cuff}}/P_0 - 1}{1 + (R_0/R_a)(P_{\text{cuff}}/P_0 - 1)}, \tag{4}$$

for $P_{\text{cuff}} \geq P_0$ (see Supplementary Figure 2, left panel). For the sake of simplicity, we define

$$\beta = \frac{R_0}{R_a} \tag{5}$$

and the two functions f and r as

$$f(x) = \begin{cases} 0, & \text{if } x \leq P_0, \\ \dfrac{x}{P_0} - 1, & \text{if } x > P_0, \end{cases} \tag{6}$$

$$r(x) = \frac{f(x)}{1 + \beta f(x)}. \tag{7}$$

Using the above definitions of the function f and r, Eq. (4) reads

$$P_v = P_0 \left\{ 1 + \left[1 + \beta f(P_a) \right] r(P_{\text{cuff}}) \right\}. \tag{8}$$

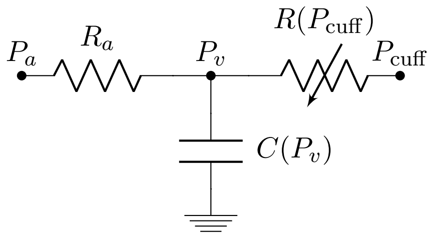

Supplementary Figure 1: Resistor-capacitance model. P_a, P_v, and P_{cuff} are the arterial, venular, and cuff pressure, $C(P_v)$ is the pressure-dependent venular compliance, R_a the total resistance upstream of venules, $R(P_{\text{cuff}})$ is the variable resistance generated by the cuff pressure.

Venular volume – venular pressure. The blood volume V in the venular compartment, represented in Supplementary Figure 2 by the capacitor $C(P_v)$ is the sum of the unstressed volume V_u and the stressed volume V_s

$$V = V_u + V_s(P_v). \tag{9}$$

The compliance varies with venular pressure P_v because it is generated by two different physiological mechanisms. At low pressures, compliance is associated with venule recruitments. At high pressures, all vessels are recruited and compliance is associated with venule wall elasticity. We can model this dependence with a step function

$$C(P_v) = \begin{cases} C_1, & \text{if } P_v \leq \bar{P}_v, \\ C_2, & \text{if } P_v > \bar{P}_v, \end{cases} \tag{10}$$

for some threshold value $\bar{P}_v$ of the venular pressure. With this assumption the total volume of the venule compartment is obtained by integrating

$$V = V_u + \int_0^{P_v} C(p)\,\mathrm{d}p. \tag{11}$$

We obtain

$$V = \begin{cases} V_u + C_1\,P_v, & \text{if } P_v \leq \bar{P}_v, \\ V_u + (C_1 - C_2)\,\bar{P}_v + C_2\,P_v, & \text{if } P_v > \bar{P}_v. \end{cases} \tag{12}$$

Inserting Eq. (8) in the above expression, we obtain an explicit relation for the venular volume V as a function of the cuff pressure P_{cuff}:

$$V(P_{\text{cuff}}) = \begin{cases} V_0 + V_1\,r(P_{\text{cuff}}), & \text{if } P_{\text{cuff}} \leq \bar{P}_{\text{cuff}}, \\ V_2 + V_3\,r(P_{\text{cuff}}) & \text{if } P_{\text{cuff}} > \bar{P}_{\text{cuff}}, \end{cases} \tag{13}$$

where

$$V_0 = V_u + C_1 P_0, \tag{14}$$
$$V_1 = C_1\,P_0\,[1 + \beta f(P_a)]\,, \tag{15}$$
$$V_2 = V_u + (C_1 - C_2)\bar{P}_v + C_2 P_0, \tag{16}$$
$$V_3 = C_2 P_0\,[1 + \beta f(P_a)]\,, \tag{17}$$

for a certain threshold value $\bar{P}_{\text{cuff}}$ of the cuff pressure P_{cuff}, which is determined by solving Eq. (8) for $P_v = \bar{P}_v$,

$$\bar{P}_v = P_0\left\{1 + [1 + \beta f(P_a)]\,r(\bar{P}_{\text{cuff}})\right\}. \tag{18}$$

Fitting the system parameters. The venular volume depends on the cuff pressure through a complex expression involving six free parameters V_u, C_1, C_2, P_0, $\bar{P}_v$, β that must be estimated by fitting the function $V(P_{\text{cuff}})$ to measured data. Computationally, it is simpler to fit $V(P_{\text{cuff}})$ as in Eq. (13) expressed with the parameters V_i, $i = 0, \ldots, 3$, P_0, and β, and then invert Eqs. (15)–(17) to determine the relevant physiological parameters.

By measuring the arterial pressure P_a:

$$C_1 = \frac{V_1}{P_0 \left[1 + \beta f(P_a)\right]}, \tag{19}$$

$$C_2 = \frac{V_3}{V_1} C_1 \tag{20}$$

$$V_u = V_0 - C_1 P_0. \tag{21}$$

The calculation of $\bar{P}_{\text{cuff}}$ and $\bar{P}_v$ is slightly more involved. Subtracting Eq. (14) from Eq. (16) and Eq. (17) from Eq. (15)

$$V_2 - V_0 = (C_1 - C_2) P_0 f(\bar{P}_v). \tag{22}$$

$$V_1 - V_3 = (C_1 - C_2) P_0 \left[1 + \beta f(P_a)\right]. \tag{23}$$

$$\tag{24}$$

We obtain

$$f(\bar{P}_v) = \frac{V_2 - V_0}{V_1 - V_3} \left[1 + \beta f(P_a)\right], \tag{25}$$

from which, using the definition of f from Eq. (6),

$$\bar{P}_v = P_0 \left\{1 + \frac{V_2 - V_0}{V_1 - V_3} \left[1 + \beta f(P_a)\right]\right\} \tag{26}$$

Comparing with Eq. (18), we obtain

$$r(\bar{P}_{\text{cuff}}) = \frac{V_2 - V_0}{V_1 - V_3} \tag{27}$$

and, finally, using the definition of r from Eq. (7)

$$\bar{P}_{\text{cuff}} = P_0 \left[1 + \left(\frac{V_1 - V_3}{V_2 - V_0} - \beta\right)^{-1}\right]. \tag{28}$$

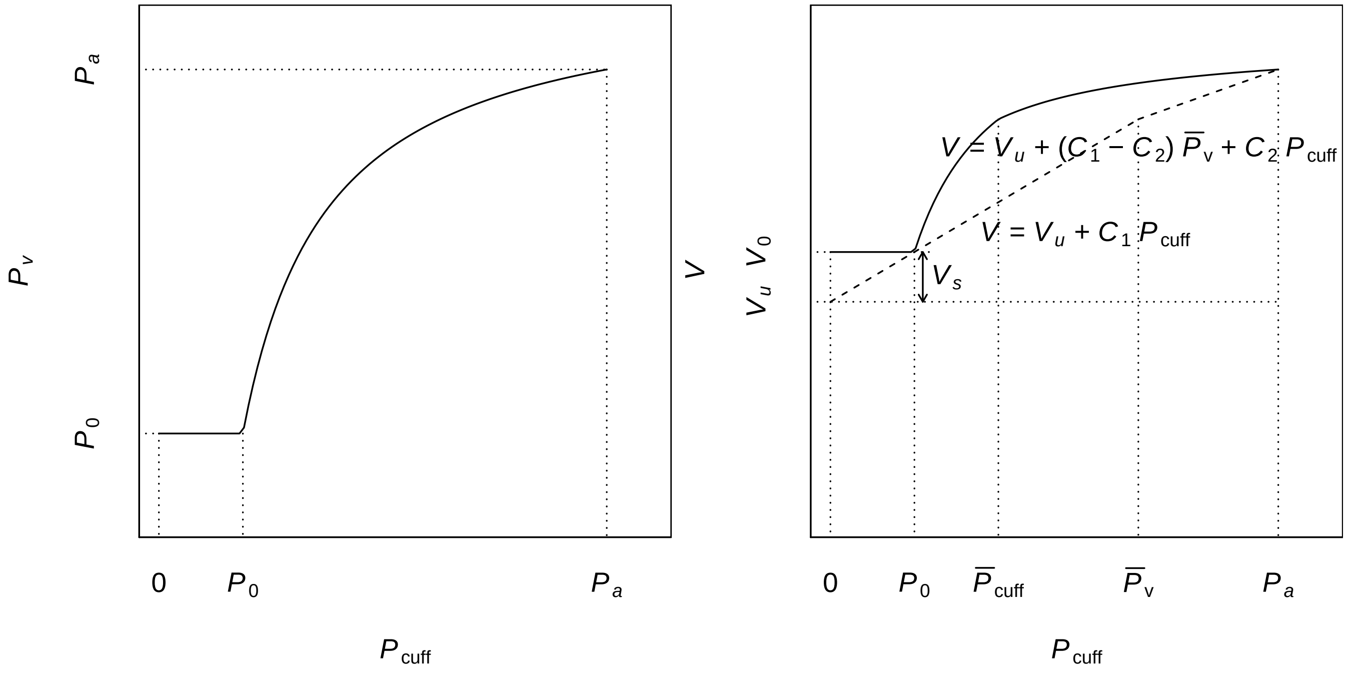

Supplementary Figure 2: Venular pressure P_v (left panel) and venular volume V (right panel) as functions of the cuff pressure P_{cuff}. P_a is the arterial pressure, P_0 the baseline venular pressure, V_u and V_s the unstressed and stressed volume, respectively, and $C(P_v)$ the venular compliance.
